# Anthrax hotspot mapping in Kenya support establishing a sustainable two-phase elimination program targeting less than 6% of the country landmass

**DOI:** 10.1038/s41598-022-24000-3

**Published:** 2022-12-15

**Authors:** John Gachohi, Bernard Bett, Fredrick Otieno, Eddy Mogoa, Peris Njoki, Mathew Muturi, Athman Mwatondo, Eric Osoro, Isaac Ngere, Jeanette Dawa, Carolyne Nasimiyu, Harry Oyas, Obadiah Njagi, Samuel Canfield, Jason Blackburn, Kariuki Njenga

**Affiliations:** 1 School of Public Health, Jomo Kenyatta University of Agriculture and Technology, Nairobi, Kenya; 2 Washington State University Global Health Program, Washington State University, P. O. Box 72938, Nairobi, 00200 Kenya; 3 Paul G, Allen School of Global Health, Washington State University, Pullman, WA99164 USA; 4 International Livestock Research Institute, Nairobi, Kenya; 5 Faculty of Veterinary Medicine, University of Nairobi, Nairobi, Kenya; 6 Kenya Zoonotic Disease Unit, Nairobi, Kenya; 7 Kenya Ministry of Agriculture, Livestock and Fisheries, Nairobi, Kenya; 8 Ministry of Health, Nairobi, Kenya; 9 Spatial Epidemiology and Ecology Research Laboratory, Department of Geography, University of Florida, Gainesville, FL 32611 USA; 10 Emerging Pathogens Institute, University of Florida, 2055 Mowry Road, Gainesville, FL 32611 USA

**Keywords:** Ecology, Diseases

## Abstract

Using data collected from previous (n = 86) and prospective (n = 132) anthrax outbreaks, we enhanced prior ecological niche models (ENM) and added kernel density estimation (KDE) approaches to identify anthrax hotspots in Kenya. Local indicators of spatial autocorrelation (LISA) identified clusters of administrative wards with a relatively high or low anthrax reporting rate to determine areas of greatest outbreak intensity. Subsequently, we modeled the impact of vaccinating livestock in the identified hotspots as a national control measure. Anthrax suitable areas included high agriculture zones concentrated in the western, southwestern and central highland regions, consisting of 1043 of 1450 administrative wards, covering 18.5% country landmass, and hosting 30% of the approximately 13 million cattle population in the country. Of these, 79 wards covering 5.5% landmass and hosting 9% of the cattle population fell in identified anthrax hotspots. The rest of the 407 administrative wards covering 81.5% of the country landmass, were classified as low anthrax risk areas and consisted of the expansive low agricultural arid and semi-arid regions of the country that hosted 70% of the cattle population, reared under the nomadic pastoralism. Modelling targeted annual vaccination of 90% cattle population in hotspot administrative wards reduced > 23,000 human exposures. These findings support an economically viable first phase of anthrax control program in low-income countries where the disease is endemic, that is focused on enhanced animal and human surveillance in burden hotspots, followed by rapid response to outbreaks anchored on public education, detection and treatment of infected humans, and ring vaccination of livestock. Subsequently, the global anthrax elimination program focused on sustained vaccination and surveillance in livestock in the remaining few hotspots for a prolonged period (> 10 years) may be implemented.

## Introduction

Although animal and human anthrax cases have declined in most developed countries due to effectiveness of control measures, African countries continue reporting a steady number of cases in humans, livestock, and wildlife, accounting for more than 75% of the 20,000–100,000 annual human anthrax cases reported globally^1^. The disease is perhaps most devastating among wildlife, with recurrent outbreaks over the last two decades associated with massive losses of endangered animal species in Namibia, Zambia, Zimbabwe, Kenya, and Tanzania^2–5^. Studies estimate that each livestock anthrax case spreads *B. anthracis* bacteria to more than 100 humans, resulting in attack rates of approximately 30% and 10% case fatality rates^5–7^. In sub-Saharan Africa (SSA), anthrax is a high priority disease targeted for active control measures that include public education, case finding, and livestock vaccination^8–10^. Studies in Kenya show that anthrax outbreaks occur in defined agro-ecological zones (AEZs) where targeted control measures can likely significantly reduce the national disease burden^5,11–14^.

We previously used a combination of retrospective data review and prospective surveillance to predict the geographic distribution of anthrax risk for southern Kenya and extrapolated this to the entire country to forecast potential future changes in these risk areas as climate change continues^13,14^. Here, we enhanced these ecological-niche modelling (ENM)-based predictions by using kernel density estimates (KDE) to identify hotspots and local indicators of spatial autocorrelation (LISA) to identify areas of greatest outbreak intensity^15,16^. The ENM approaches used were validated using prospectively collected anthrax outbreak data. The outcome was a risk map that stratified the country into three risk profiles: hotspots, high-risk, and low risk areas plus a qualitative map showing spatial association in administrative wards to enhance potential for targeted interventions by the government. Subsequently, we modeled the impact of vaccinating livestock in the identified hotspots as a national control measure.

## Materials and methods

### Data sources

This study builds on two datasets; 666 livestock anthrax outbreaks collected over 60 years (1957–2017) by the Kenya Directorate of Veterinary Services (KDVS), and 13 reported anthrax outbreaks we investigated between 2017 and 2018^11,13^. These datasets were combined with data from targeted active anthrax surveillance we conducted in 2019–2020 (*see below*) to define anthrax suitable areas in Kenya, including hotspots, and subsequently assessed effectiveness of livestock vaccination as a control strategy.

### Targeted active surveillance-collected anthrax data, 2019–2020

Active anthrax surveillance was conducted for 12 months between 2019 and 2020 in randomly selected areas to ensure representation of all AEZs of the country. AEZs are land units defined based on the patterns of soil, landforms and climatic characteristics. Kenya has seven AEZs that include agro-alpine, high potential, medium potential, semi-arid, arid, very-arid and desert. In 2013, Kenya devolved governance into 47 semi-autonomous counties that are subdivided into 290 subcounties which are in turn divided into 1450 administrative wards, the smallest administrative units in the country. Using a geographic map that condensed Kenya into five AEZs; agro-alpine, high potential, medium potential, semi-arid, and arid/very arid zones, we randomly selected 4 administrative sub-counties from each AEZ (N = 20). To increase geographic spread of the study and enhance detection of anthrax outbreaks, we surveilled the larger administrative county (consisting of 20 to 45 administrative wards) where the randomly selected sub-counties were located. As shown in Fig. S1, we ultimately carried out the active anthrax surveillance in 18 counties, containing 523 administrative wards, the latter being used for measuring spatial association (*see below*).

We conducted the surveillance between April 2019 and June 2020, through 523 animal health practitioners (AHPs), one in each ward, after intensive training to identify anthrax using a standard case definition, and to collect and electronically transmit the data weekly using telephone-based short messaging system (SMS) to a central server hosted by KDVS. Regarding case definition, any livestock death classified as anthrax through clinical or laboratory diagnosis was considered an anthrax event. Using standard guidelines issued by the KDVS, a clinical diagnosis was made by the AHPs across the country as an acute cattle, sheep or goat disease characterized by sudden death with or without bleeding from natural orifices, accompanied by absence of rigor mortis. Further, if the carcass was accidentally opened, failure of blood to clot and/or the presence of splenomegaly were included. In pigs, symptoms included swelling of the face and neck with oedema. A laboratory confirmed anthrax was diagnosed using Gram and methylene blue stains followed by identification of the capsule and typical rod-shaped *B. anthracis* in clinical specimens that the AHPs submitted to the central or regional veterinary investigation laboratories in Kenya. One case of anthrax in either species was considered an outbreak.

During the surveillance, the programmed server sent prompting texts directly to the AHPs' cell phones every Friday of each week for the 52 weeks. The AHPs interacted with the platform by responding to prompting questions sent via SMS to their telephones. Data were securely stored in an online encrypted platform which was subsequently downloaded into Ms Excel for analysis. This surveillance detected 119 anthrax outbreaks, whose partial data were used to model effects of climate change on future anthrax distribution in Kenya^14^. Here, we integrated these active surveillance data with other datasets to conduct detailed ENM and kernel-smoothed density mapping with a goal of refining suitable anthrax areas including crystalizing hotspots in the country.

### Anthrax outbreak incidence per livestock population by county

We knew the total number of livestock per county and wards by species for the active surveillance period. The counties represented the level of disease management including vaccine distribution while the wards within counties represented the modeling unit for targeting control. Therefore, we estimated the outbreak incidence as the total number of outbreaks per livestock species per 100,000 head of that species.

### Ecological niche modeling and validation

We used boosted regression tree (BRT) algorithm as previously published^13^. In those studies, we estimated the geographic distribution of anthrax in southern Kenya using 69 spatially unique outbreak points (thinned from the 86 outbreaks in the records) and 18 environmental variables resampled to 250 m resolution. In this study, the final experiments were run with a learning rate (lr) = 0.001, bagging fraction (br) = 5, and maximum tree = 2500. We then mapped anthrax suitability as the mean output of the 100 experiments and the lower 2.5% and upper 97.5% mapped as confidence intervals. We determined variable contribution and derived partial dependence as previously described^13^. As BRTs are a random walk and each experiment randomly resamples training and test data, it was necessary to repeat those outputs along with the map predictions.

Here, our goal was to evaluate the BRT models built with records data from 2011 to 2017 data and use the *predict* function to calculate model accuracy metrics using the 2017–2020 outbreaks as presence points and the sub-counties reporting zero outbreaks during the 2019–2020 active surveillance period as absence points. The model of southern Kenya was projected onto all of Kenya using climate variables clipped to the whole of Kenya. We tested the BRT models in two ways; first, evaluating 2011–2017 data models with holdout data using a random resampling and multi-modeling approach. Here, we report the area under curve (AUC) for each of the original training/testing split into the 69 historical points and the 2017–2020 data serving as independent data, the latter representing true model validation. Second, to determine the total percentage of surveillance data predicted and map areas of anthrax suitability to compare with kernel density estimates (see below), we produced a dichotomized map using the Youden index cutoff^17^ following Otieno et al.^14^.

### Outbreak concentrations from kernel density estimation (KDE)

To describe the spatial concentration of reported outbreaks, we calculated descriptive spatial statistics, including the spatial mean, standard distance, and standard deviational ellipse of outbreak locations from the prospective surveillance dataset following Blackburn et al.^18^ These spatial statistics help to differentiate the geographic focus (spatial mean) and dispersion of outbreak reports from year to year and across the sampling period. We then conducted kernel density estimation (KDE) to visualize the concentration of anthrax outbreaks per square kilometer per year and across the study period^18^. We used the *spatstat* package for all KDE analyses using the quadratic kernel function^19^:

$$f\left( x \right) = \frac{1}{{nh^{2} }} \mathop \sum \limits_{i = 1}^{n} K\left( {\frac{{x - X_{i} }}{h}} \right)$$

where *h* is the bandwidth, x*-X*_*i*_ is the distance to each anthrax outbreak *i.* Finally,* K* is the quadratic kernel function, defined as:

$$K\left( x \right) = \frac{3}{4}\left( {1 - x^{2} } \right), \left| x \right| \le 1$$

$$K\left( x \right) = 0,x > 1$$

This function was employed to estimate anthrax outbreak concentration across space using each outbreak weighted as one. We calculated the bandwidth (kernel) using *h*_*opt*_ that uses the sample size (number of outbreaks) and the standard distance to estimate bandwidth. Finally, we estimated bandwidth for each year and then averaged them to apply the same fixed bandwidth for each year under study in Q-GIS version 3.1.8. The resulting outputs were map surfaces representing the spatial concentrations of outbreaks across the country per 1 km^2^ for each study year and all study years combined. For this study, we used the cutoff criteria of Nelson and Boots^19^ to identify outbreak hotspots as areas with density values in the upper 25%, 10%, and 5% of outbreak concentrations. The analyses identified these areas by year (2017–2020) and for all surveillance years combined.

### Local spatial clustering at the ward level

#### Anthrax outbreak incidence per livestock species

The ENM and KDE-derived maps provide a first estimate of potential risk and outbreak concentration, respectively. We were also interested in estimating anthrax outbreak intensity relative to livestock populations at a local level. For the active surveillance period, we knew the total number of outbreaks per ward (the smallest administrative spatial unit) by livestock species. For this two-year period, we estimated the ward-level outbreak incidence as the total number of outbreaks per livestock species per 10,000 head of that species. To estimate livestock population per ward, we extracted the values in the raster file of the areal weighted gridded livestock of the world data using the zonal statistic routine in Q-GIS version 3.1.8, into the polygon consisting of all pixels per ward as the total population^19,20^. We calculated outbreak incidence as the number of outbreaks per ward cattle population per 10,000 cattle for each administrative ward. We limited this analysis to those 18 counties participating in the active surveillance study (Fig. S1), as we could appropriately assume any ward with no reports was a ‘true zero’ for the estimation. Given that most reported outbreaks were in domestic cattle (see results below), we here report those results involving cattle alone. Given the overall high number of wards and the high number of wards without outbreaks, we performed the empirical Bayes smoothing and spatial Bayes smoothing routines in GeoDa version 1.12.1.161 to reduce the variance in anthrax incidence estimates^20,21^. To evaluate smoothing routine performance, we box plotted rates per ward and selected the method with the greatest reduction in outliers^21^. Smoothed rates were mapped as choropleth map in Q-GIS version 3.1.8 using the four equal area bins.

### Spatial cluster analysis

We used Local Moran’s I^16^ to test for spatial cluster of livestock anthrax in cattle using the smoothed outbreak incidence estimates. The Local Moran’s I statistic tests whether individual wards are part of spatial cluster, like incidence estimates surrounded by similar estimate (high-high or low-low) or spatial outliers where wards with significantly high or low estimates are surrounded by dissimilar values (high-low or low–high). The local Moran’s I is written as^16^:

$$I_{i} = Z_{i} \sum W_{ij} Z_{j}$$

where *I*_*i*_ is the statistic for a ward *i,* Z_*i*_ is the difference between the incidence at *i* and the mean anthrax incidence rate for all of wards in the study, *Z*_*j*_ is the difference between anthrax risk at ward *j* and the mean for all wards. *W*_*ij*_ is the weights matrix. In this study, the 1^st^ order queen contiguity was employed. Here, *W*_*ij*_ equals 1/n if a ward shared a boundary or vertex and 0 if not. For this study, Local Moran’s I was performed on the wards using 999 permutations and *p* = 0.05 using GeoDa version 1.12.1.161.

### Assessing effectiveness of cattle vaccination in burden hotspots

As a first estimate of how we might scale up livestock anthrax vaccination efforts in Kenya, we slightly adjusted a simple published anthrax outbreak simulation model in a cattle population. For this study we applied an early mathematical approach of Funiss and Hahn^22^ to simulate anthrax at the ward level. While other recent models are available^23,24^, these are difficult to parameterize or require time series data we could not derive with the surveillance approach in this study. Like the more recent models, Funiss and Hahn^22^ assumed anthrax transmission was driven by cattle accessing spore-contaminated environments. Here the proportion of infected cattle each day depended on the population of susceptible animals in the population and probability of getting infected. This probability depends on environmental contamination (“*a*”), and a fraction of anthrax carcasses in the environment on a day (“*f,”)*. Each day, the newly infected cattle are transferred to an incubation period vector, “*d,”* waiting to die following a probability *“p”*. In this model, all infected animals, “*n,”* die following the incubation periods given by the vector, “*p”*, in which *p*_*i*_ is the probability of a cow dying *i* days after the infection. Following death, the cattle are transferred to a carcass state, providing a direct infection source to the susceptible cattle via environmental contamination. Environmental contamination “*a,”* is therefore defined as the number of spores ingested by an animal in a day. This environmental contamination depends on spores from carcasses and an assumed spore decay rate γ^22^.

The complete set of difference equations with a daily time step is given by:

$${\text{S}}_{(t + 1)} = {\text{S}}_{(t)} - {\text{ S}}_{(t)} *\left( {{1} - {\text{e}}^{{ - \left( {{\text{a}}_{t} + \gamma {\text{f}}_{{{\text{t}} + 1}} } \right)}} } \right)$$

$${\text{I}}_{(t + 1)} = {\text{I}}_{(t)} + {\text{ S}}_{(t)} *\left( {{1} - {\text{e}}^{{ - \left( {{\text{a}}_{{\text{t}}} + \gamma {\text{f}}_{{{\text{t}} + {1}}} } \right)}} } \right)$$

where the expression $\left( {{1} - {\text{e}}^{{ - \left( {{\text{a}}_{t} + \gamma {\text{f}}_{{{\text{t}} + 1}} } \right)}} } \right)$ denotes the probability of an animal becoming infected and a_*t*_ + γf_*t*+*1*_ is the mean number of spores ingested by a cow in a day. The equation for environmental contamination, a, is given by:

$${\text{a}}_{t + 1} {-}{\text{a}}_{{\text{t}}} = \alpha {\text{a}}_{{\text{t}}} + \beta {\text{c}}_{{{\text{t}} + {1}}}$$

The newly infected animals die after a certain number of days. The distribution of incubation periods is given by the vector, *p*. On each day, the new cases are placed in a due-to-die vector, *d,* and when they die, they are subsequently moved down one step to fresh carcasses, *f*_*t*_. The fresh carcasses provide a direct source of infection to the susceptible cattle via the ‘fresh carcass term’, γ. These carcasses decay or are scavenged or disposed by man. The equation expressing the disseminating carcasses, c, is:

$${\text{C}}_{t + 1} - {\text{c}}_{t} = {\text{f}}_{t + 1} - \delta {\text{c}}_{t}$$

The model parameters variables are provided in Table 1 and are similar to those used by Funiss and Hahn^22^ to generate a standard run. We ran the model for one year and extrapolated to cattle population in the identified hotspot wards.

**Table 1:** Model parameters and variables.

Parameter description	Symbol	Value
Decay rate	α	0.01
Fresh carcass term providing a direct source of infection to the susceptible cattle	γ	0.001
Rate of increase by spores disseminated from carcasses	β	0.001
Carcass half life	δ	0.6
Distribution of incubation periods	p	0.2, 0.4, 0.2
Initial susceptible	S_*o*_	120
Initial infected	I_*o*_	0
Initial fresh carcass	f_*o*_	1
Environmental contamination	a_*o*_	0
Initial number of carcasses in the field	c_*o*_	0

## Results

### Anthrax incidence—county level

Figure S2 shows the point locations of livestock anthrax outbreaks obtained from field characterization of reported outbreaks (2017–2018) and active surveillance effort (2019–2020). We recorded 119 anthrax outbreaks in 2019–2020, with an overall anthrax incidence of 0.31 outbreaks per 100,000 animals (95% CI, 0.25, 0.38) per year. The species-specific incidence was highest among cattle and pigs, with more than one outbreaks per 100,000 animals, while sheep and goats had the lowest incidence of less than 0.1 outbreaks per 100,000 animals (Table 2). There was no reported anthrax outbreak in donkeys. Out of the 171 animals affected in these 119 outbreaks, 88% (ratio of 7:1) were cattle with a mean number of 1.13 animals per outbreak (median = 1.0).

**Table 2:** Species-specific incidence (95% CI) of anthrax outbreaks ranked per 100,000 animals per year.

Species	Incidence	95%CI	Number of animals affected
Cattle	1.73	1.48, 2.03	151
Pigs	1.4	0.20, 9.94	1
Camels	0.16	0.06, 0.43	4
Sheep	0.08	0.04, 0.16	8
Goats	0.04	0.02, 0.10	7

### 2019–2020 outbreaks by agroecological zones in Kenya

Of the 18 study counties, 12 (67%) reported at least one outbreak, with the highest incidence reported in the agriculturally productive Vihiga, Bomet, and Murang'a counties. The drier agriculturally unproductive counties of Kitui, Garissa, and Marsabit reported the lowest incidence. We recorded more outbreaks in the high potential (44%), medium potential (27%) and agro-alpine (humid) (20%) AEZs, whereas the arid and semi-arid AEZs accounted only 9% of the outbreaks (Table 3).

**Table 3:** Proportion and incidence of anthrax outbreaks by agroecological zones.

Agroecological zone	Anthrax outbreaks (%)	Incidence per 10,000 animals per year	95% CI
High potential	44	46	35, 61
Medium potential	27	35	25, 49
Agro-alpine	20	44	12, 155
Semi arid	8	2.8	2.0, 5.0
Arid and very arid	1	0.05	0.0, 0.3

Of the 12 counties that reported outbreaks, four accounted for 74% of all outbreaks. Within the 523 smallest administrative units (wards) where active surveillance occurred, outbreaks were reported in 49 (9.4%) wards.

### Anthrax risk mapping

**Figure 1:**
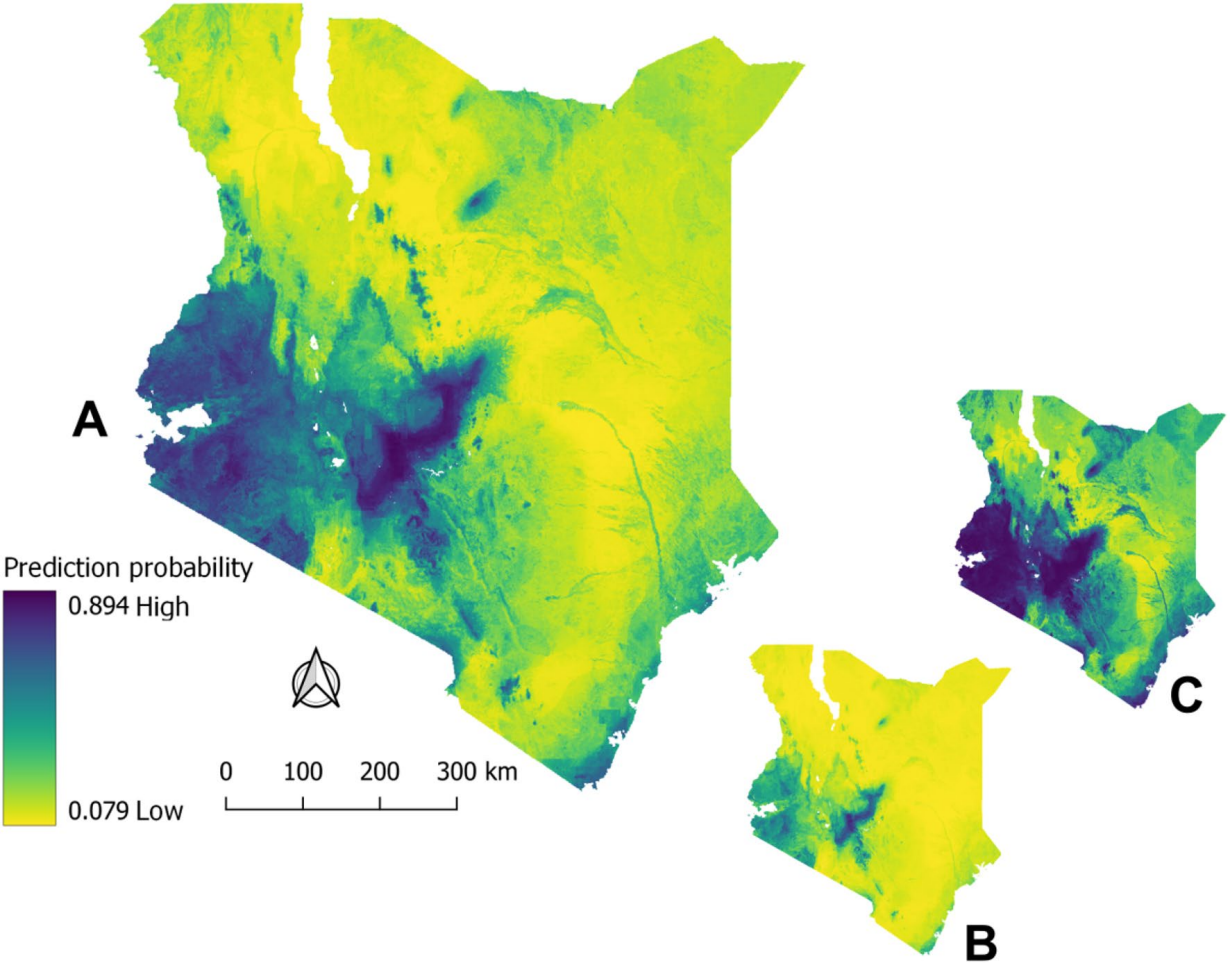
Panel (**A**): Extrapolated geographic prediction for anthrax to the whole of Kenya landscape using southern Kenya-derived 100 BRT experiments. (**B**) shows the lower 2.5% and (**C**), the upper 97.5% confidence intervals. This figure was generated using R software version 4.2.2. at http://cran.r-project.org.

Using ENM, we predicted the regions covering the southwestern area of Kenya extending into a belt running in a northeastern direction towards central highlands as having the highest suitability for the disease (Fig. 1). The suitable region extended to the western highlands and the Lake Victoria basin bordering Uganda, and a narrow strip along the Indian Ocean coast. In contrast, the country's semi-arid and arid lands in the northern and southeastern regions showed very low suitability for anthrax (Fig. 1). The accuracy of this prediction model was confirmed as described in supplementary text S1.

Boosted regression tree (BRT) modeling identified cattle density, soil pH, soil clay and organic content, rainfall during the wettest month, and the length of the dry season as risk factors of disease occurrence (Fig. S3).

### Combined hotspot and risk mapping

**Figure 2:**
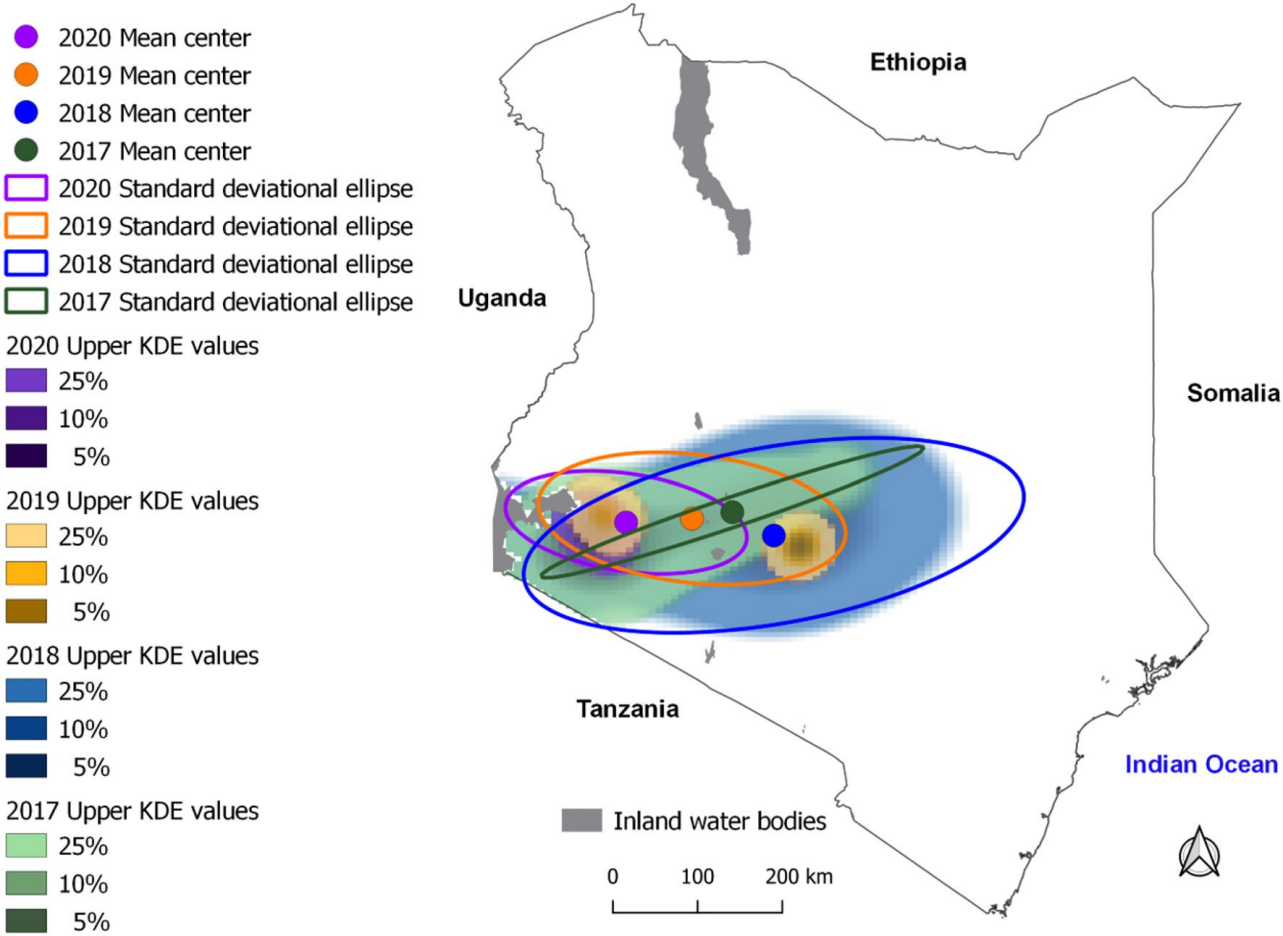
Multiyear kernel density derived hotspots and standard deviational ellipses shown as ellipsoid shapes. The ellipses illustrate the directionality of anthrax outbreaks across years. Note the size, shape, and overlap of ellipses in different years (colours correspond to the study years). The kernel density defines the spatial distribution of field characterization and active surveillance anthrax outbreaks (2017–2020) densities. This figure was generated using Q-GIS software version 3.1.8 at http://qgis.org.

The KDE identified an overall concentration of anthrax risk in the same southwestern region of the country with some inter-annual variability (Fig. 2). The 2017 and 2018 periods had more easterly spatial spread when compared to 2019 and 2020 periods. While the 2017 directional trend of the outbreaks narrowed from southwestern to central Kenya, standard deviational ellipses identified 2018 as the year with the broadest dispersion or more considerable outbreak extent and 2020 as the year with the greatest concentration of outbreaks in a smaller area (Fig. 2).

**Figure 3:**
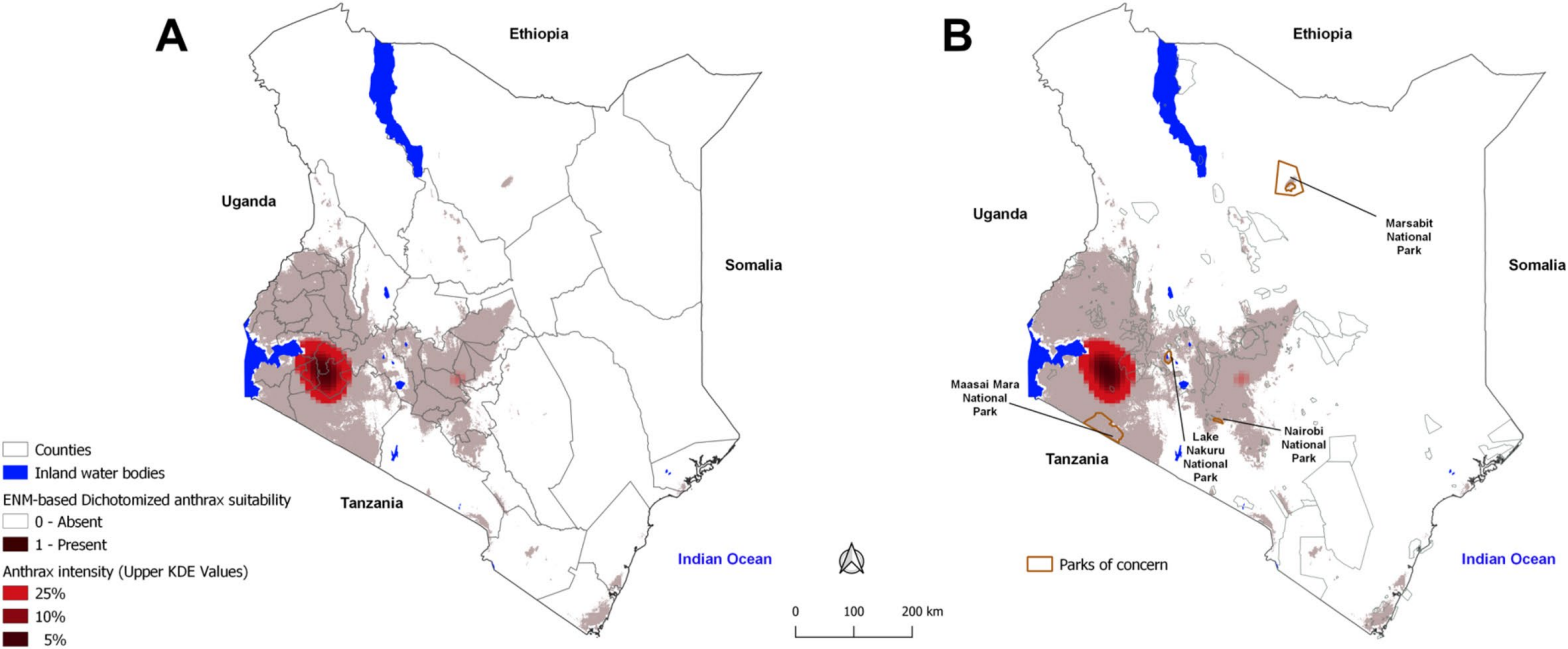
Kernel density derived hotspots (red to dark red areas) showing the spatial distribution of active surveillance anthrax outbreak (2018–2020) densities overlaid over ecological niche modelled (ENM) areas dichotomized as suitable or not suitable (grey versus white) employing historical records (2011–2017). The ENM was independently evaluated using active surveillance anthrax outbreaks. Left: showing county boundaries. Counties are responsible for implementing local disease control measures; Right: map of Kenya showing wildlife conservation parks with four parks of concern primarily falling in the suitable areas. These figure was generated using both R software version 4.2.2. at http://cran.r-project.org and Q-GIS software version 3.1.8 at http://qgis.org.

A combined hotspot and risk map, generated by overlaying the KDE-derived hotspots and the predicted risk map developed by ENM is shown in Fig. 3. We defined two hotspots indicated by the red shading in Fig. 3; the larger one in southwestern region spanning seven counties, centered around Bomet county and extending to Narok, Kisii, Nyamira, Homa Bay, Kisumu, and Kericho counties, and a smaller one in central Kenya spanning Muranga and Kirinyaga counties (a total of 9 counties) with 79 administrative wards (Fig. 3A). The nine hotspot counties accounted for 62% (82/132) of all 2017–2020 anthrax outbreaks identified through outbreak characterization and active surveillance studies. The broader anthrax risk region consisted of 30 counties with 1043 administrative wards (red and grey shaded in Fig. 3A). Out of these 30 counties, the nine hotspot counties host 9% of the country`s livestock population, whereas the other 21 counties host 21% of the livestock population. Stall-fed small-scale commercial livestock production systems with intensification and higher human density serving as a source of milk markets primarily characterize the hotspot counties. The rest of anthrax suitable regions largely practice mixed crop-livestock system that integrate the growing of food and cash crops with a semi-stall feeding type of livestock production that combines or alternates between stall and free grazing systems. The remaining 17 counties consisting of 407 wards hosts 70% of the country's livestock mostly under pastoralist production system in areas of low or no risk of anthrax.

Over 65% of all wildlife conservation areas are in the anthrax suitability region (Fig. 3B), including the important Maasai Mara, Lake Nakuru, and Nairobi national parks that contain large populations of endangered wildlife species.

### Local spatial clustering at the ward level

While the county level analysis helped to identify general agroecological zones, we were also interested in the localized patterns of high anthrax incidence in those wards reporting disease, as these analyses could identify primary areas for livestock vaccination and public health campaigns. An examination of smoothed rates revealed empirical Bayes as best reducing outliers (not shown). The smoothed incidence of outbreaks per cattle population at the ward level is mapped in Fig. S4. Local Moran’s I based clusters and outliers are mapped in Fig. S4. High-High clusters and High-Low spatial outliers were identified in each of southwestern and southcentral Kenya, with an additional High-Low outlier in the north. Wards of High-High clustering or High-Low spatial outliers identified in the Local Moran’s I analysis corresponded with KDE-defined concentration hotspots.

### Impact of livestock vaccination at hotspots as a control measure

Assuming anthrax endemicity with outbreaks running for a year in a naïve cattle population located in the 79 hotspot administrative wards, we determined anthrax would kill 260 of the 1,162,662 cattle population in these wards. However, vaccinating 90% of the cattle protect approximately 23,400 humans from anthrax exposure.

## Discussion

Using data from a year-long active anthrax surveillance in randomly selected sub-counties covering all AEZs of Kenya, we first validated a recent risk map and then applied both KDE and LISA to spatially identify hotspots and crystalize local clusters of high and low outbreak intensity using administrative wards while at the same time appropriately modeling autocorrelations with the neighbouring wards. The combined approaches identified the western and southwestern regions of the country, narrowing eastwards into the central Kenya highlands as the anthrax suitable belt with a high consistency of temporal occurrence of the disease. Of Kenya's 1450 administrative wards, the study identified high burden hotspots in only 79 wards covering 5.5% of the country landmass and hosting 9% of the country’s 13 million cattle population. These findings supported disease clustering in certain ecological foci within high agricultural AEZs, characterized by high cattle density, favorable soil types, water and humidity, temperature, and vegetation. Similar anthrax permissive ecosystems were described in United States, Europe and Australia, where enhanced surveillance and control programs were implemented leading to highly successful containment of the disease^25–27^. Given that *B. anthracis* persists in the environment for decades^28^, mapping disease hotspots provides policy-makers with critical data for targeting control programs that must be sustained for prolonged durations^29^.

This Kenyan risk map we have developed enables the government to develop and implement targeted, and thus cost-effective prevention and control program for anthrax using a One Health approach that minimizes human diseases through surveillance and timely intervention in animals. The map can also be used by scientists to target studies on maintenance of *B. anthracis,* effectiveness of various intervention approaches, and the role of social-cultural factors in animal-to-human infections. Regionally, a similar approach can be used across countries in sub-Saharan Africa where anthrax is a high priority zoonotic disease; thus needing refined anthrax risk maps that can enhance rolling out of cost-effective and sustainable anthrax prevention and control strategies^8–10^.

The adopted global anthrax elimination program entail sustained vaccination and surveillance of livestock, covering close to 100% of the animals, in identified hotspots for a prolonged period (> 10 years), followed by sustained surveillance for a continuously long duration^30–32^. Recent success of this global approach was demonstrated in Azerbaijan where an upsurge of human anthrax cases followed premature cessation of livestock vaccinations, associated with the collapse of the former Soviet Union^32^. As the Azerbaijani animal health system rebuilt following independence, human case numbers dropped as livestock vaccination was reintroduced in 2007^32^. Interestingly, a recent pilot study at Dien Bien province in Vietnam showed sustained vaccination of at-risk livestock at a low coverage of ~ 30% reduced human and livestock anthrax incidence; rates increased in both populations in years following decreases in livestock vaccination rates (Luong et al. 2022 under review).

For Kenya and other low- and medium-income countries where anthrax is endemic in sizable areas of the country, a phased approach to prevention and control may be more economically viable. The first phase, designed to shrink hotspots should be focused on enhanced animal and human surveillance in identified hotspots, followed by rapid response to outbreaks anchored on public education, proper carcass disposal, detection and treatment of infected humans, and targeted vaccination of livestock. During this phase, other interventions that should be rolled out include; (i) regular one-health capacity strengthening campaigns while increasing cross-disciplinary communication among health practitioners and laboratory diagnosticians in both animal (livestock and wildlife) and human sides^33–35^, (ii) improved community health that addresses the socio-cultural and economic determinants of anthrax spillover to humans^36–38^, and (iii) enhanced syndromic surveillance in wildlife^39^. Passive surveillance and response must also be maintained in other anthrax suitable areas that are not burden hotspots, and at low-risk areas. The second and final phase of national anthrax control program would entail embracing the global anthrax elimination program of sustained vaccination of all livestock in the remaining few hotspots for a prolonged period, accompanied by intensive surveillance that persist beyond the vaccination period.

Our suggested 2-phase approach to eliminating anthrax was informed by our modelling work we did in the current study that assessed the efficacy and cost effectiveness of cattle vaccination program in the Kenyan anthrax hotspot belt we identified. The study focused on cattle vaccination since our previous and other studies showed > ninefold higher anthrax outbreaks involving cattle when compared to other livestock, and > 95% of human anthrax cases in various studies were associated with cattle anthrax^11,40,41^. Our cattle vaccination effectiveness study demonstrated that annual vaccination of 90% of the approximately 1.2 million cattle population in the hotspots, an expensive undertaking in hotspot administrative wards, reduced more than 23,000 human exposures. Our modelling used the previous approach by Funiss and Hahn^22^ to assume that all infected cattle die, which is likely an unrealistic assumption given recent serological studies of anthrax in the region and elsewhere that suggest chronic but non-fatal exposure to *B. anthracis*^29,42–44^ particularly in bovid species. This approach also excludes the role of environmental drivers and spore populations, which impact outbreak intensity^23,24^.

The anthrax hotspots in Kenya, located in agriculturally productive regions, had characteristic risk factors including high cattle density, permissive climatic variables, and fertile soils with properties suitable for *B. anthracis* spore viability and persistence. These regions practiced intensified livestock husbandry systems aiming for higher milk production and access to high-value markets. High cattle density in these regions could escalate pathogen exposure from greater soil contact per unit of land. Nevertheless, consistent with other studies, changes in the environment in response to weather extremes linked to factors such as long dry seasons and excessively wet months appear to trigger outbreaks as reported elsewhere^29^. These findings suggest that using green-indices signatures from remotely sensed vegetation indices from Moderate Resolution Imaging Spectroradiometer satellite data as an early warning system to identify anthrax periods in Africa similar to other regions is possible^45^. A vegetation index quantifies vegetation biomass and/or plant vigor in response to soil and atmospheric moisture for each pixel in a remote sensing image. Still, variations in concentrations of *B. anthracis* in the soil could play a role in response to weather extremes though this needs further research. Other reports indicate that an increase in host susceptibility resulting from these weather extremes and social or reproductive stressors trigger anthrax outbreaks, especially in wildlife^46^. Indeed, over 65% of all wildlife conservation areas fall in the anthrax suitability region hosting large populations of endangered wildlife species highlighting the need for improved wildlife disease surveillance for greater wildlife conservation that guarantees sustained biodiversity but also foreign exchange earnings.

This study has several strengths. First, we reinforced the study by evaluating the risk map with spatially independent data. Model evaluation is critical, especially when generating maps for guiding surveillance and disease management and using data sets subject to reporting bias, such as the historical data set comprising passively collected data^47^. Second, the independent dataset employed a longitudinal design using a large nationally representative cohort of 523 administrative units generating close to 27,000 observations covering all AEZs. The nil loss to follow-up and the high weekly response rates in collecting the longitudinal data contributed to the data credibility and minimized the reporting bias. Nevertheless, we were limited in the time taken in data collection since we developed the risk model using data spanning six years and evaluated the model with data covering four years.

In conclusion, our findings support an economically viable first phase of anthrax control program in low-income countries where the disease is endemic, that is focused on enhanced animal and human surveillance in burden hotspots, followed by rapid response to outbreaks anchored on public education, detection and treatment of infected humans, and ring vaccination of livestock. Subsequently, the global anthrax elimination program focused on sustained vaccination and surveillance in livestock in the remaining few hotspots for a prolonged period (> 20 years) can be implemented.

## Supplementary Information

Supplementary Figure S1.

Supplementary Figure S2.

Supplementary Figure S3.

Supplementary Figure S4.

Supplementary Information 1.

## Data Availability

The datasets generated and/or analysed during the current study are not publicly available since they belong to the Directorate of Veterinary Services under the Government of Kenya but are available from the corresponding author on reasonable request.
