# Supplementary material for: Anthrax hotspot mapping in Kenya support establishing a sustainable two-phase elimination program targeting less than 6% of the country landmass
Source: Sci Rep. 2022 Dec 15;12:21670. doi: 10.1038/s41598-022-24000-3 (PMC9755300; doi:10.1038/s41598-022-24000-3)
Supplement: Supplementary file 1 — Supplementary Figure S1. [file 41598_2022_24000_MOESM1_ESM.docx]

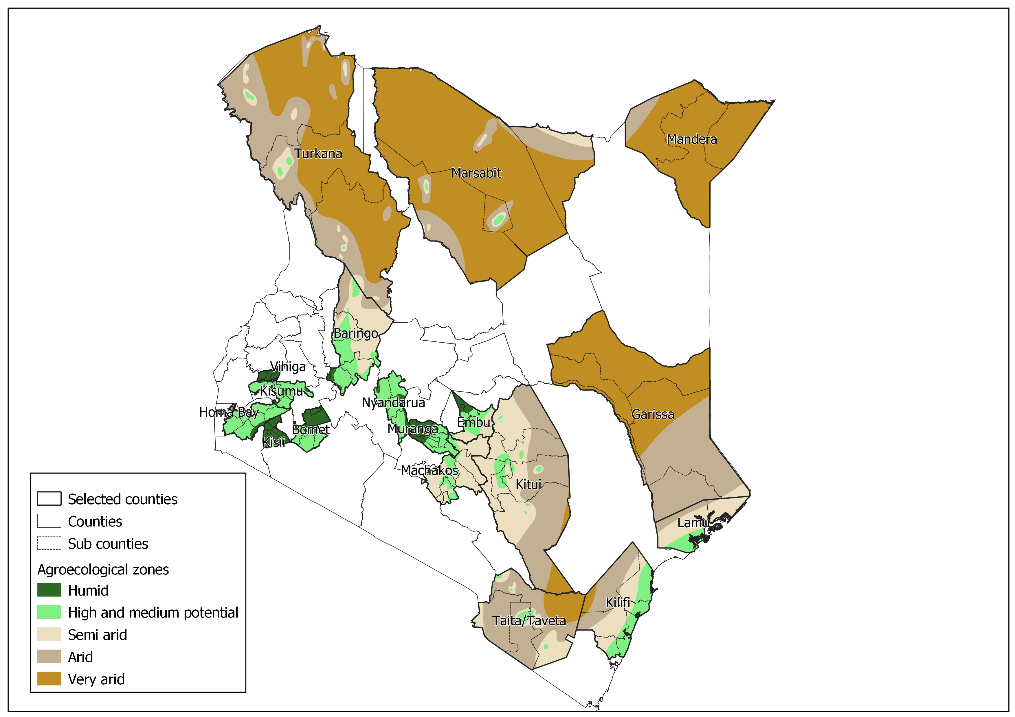


***Fig S1: Distribution of the randomly selected 18 counties (hosting the 115 sub-counties and 523 wards) overlaid on the five AEZs.*** *This figure was generated using Q-GIS software version 3.1.8 at* [*http://qgis.org*](http://qgis.org)
