# Supplementary material for: Anthrax hotspot mapping in Kenya support establishing a sustainable two-phase elimination program targeting less than 6% of the country landmass
Source: Sci Rep. 2022 Dec 15;12:21670. doi: 10.1038/s41598-022-24000-3 (PMC9755300; doi:10.1038/s41598-022-24000-3)
Supplement: Supplementary file 2 — Supplementary Figure S2. [file 41598_2022_24000_MOESM2_ESM.docx]

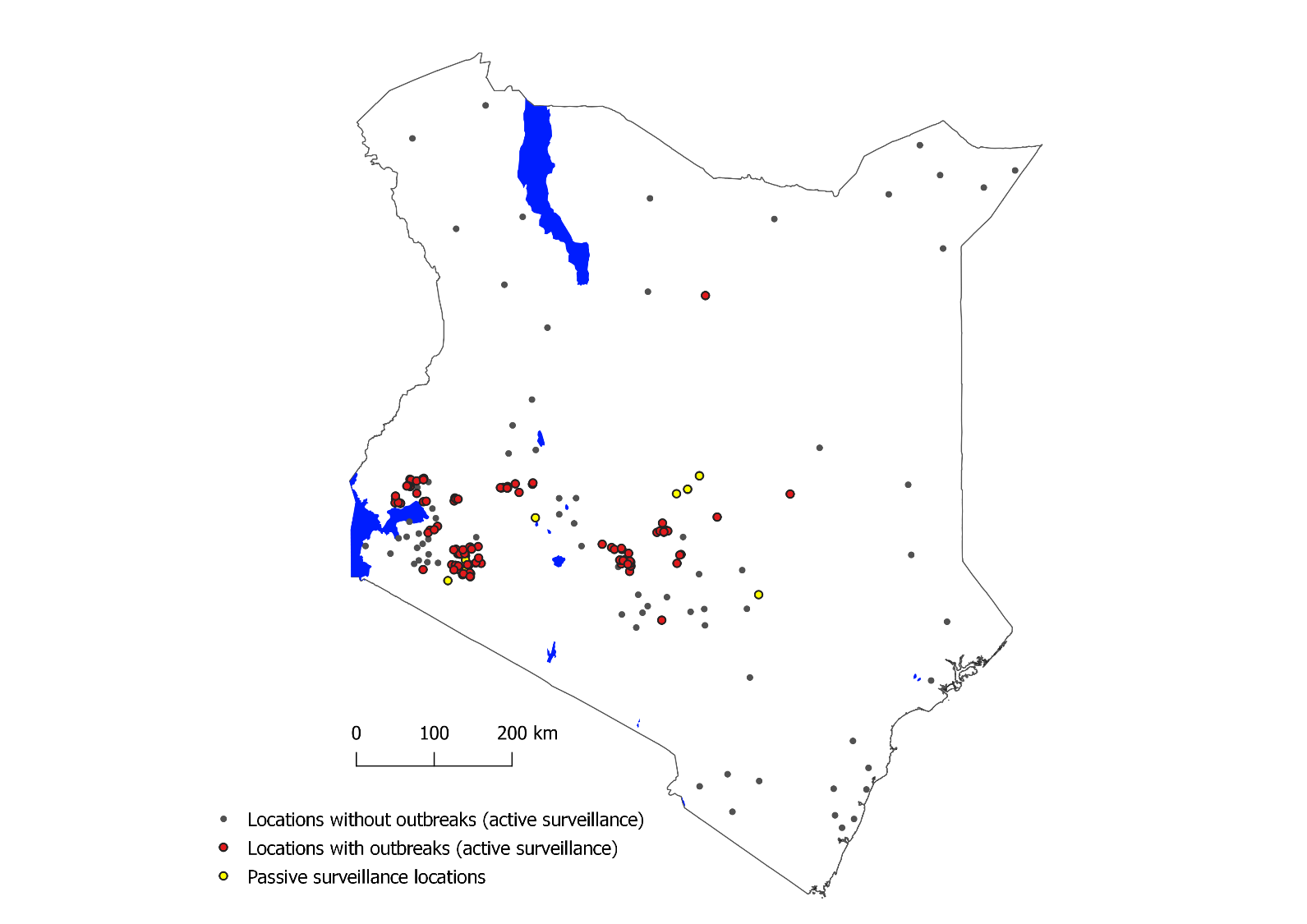


***Figure S2. Distribution of livestock anthrax outbreak locations from field characterization of reported outbreaks (2017, 2018; yellow dots) and active surveillance effort (2019, 2020; red dots). Absence points represent the centroid of each subcounty enrolled in the study that did not report anthrax during the active surveillance period.*** *This figure was generated using R software version 4.2.2. at* [*http://cran.r-project.org*](http://cran.r-project.org)*.*
