# Supplementary material for: Anthrax hotspot mapping in Kenya support establishing a sustainable two-phase elimination program targeting less than 6% of the country landmass
Source: Sci Rep. 2022 Dec 15;12:21670. doi: 10.1038/s41598-022-24000-3 (PMC9755300; doi:10.1038/s41598-022-24000-3)
Supplement: Supplementary file 3 — Supplementary Figure S3. [file 41598_2022_24000_MOESM3_ESM.docx]

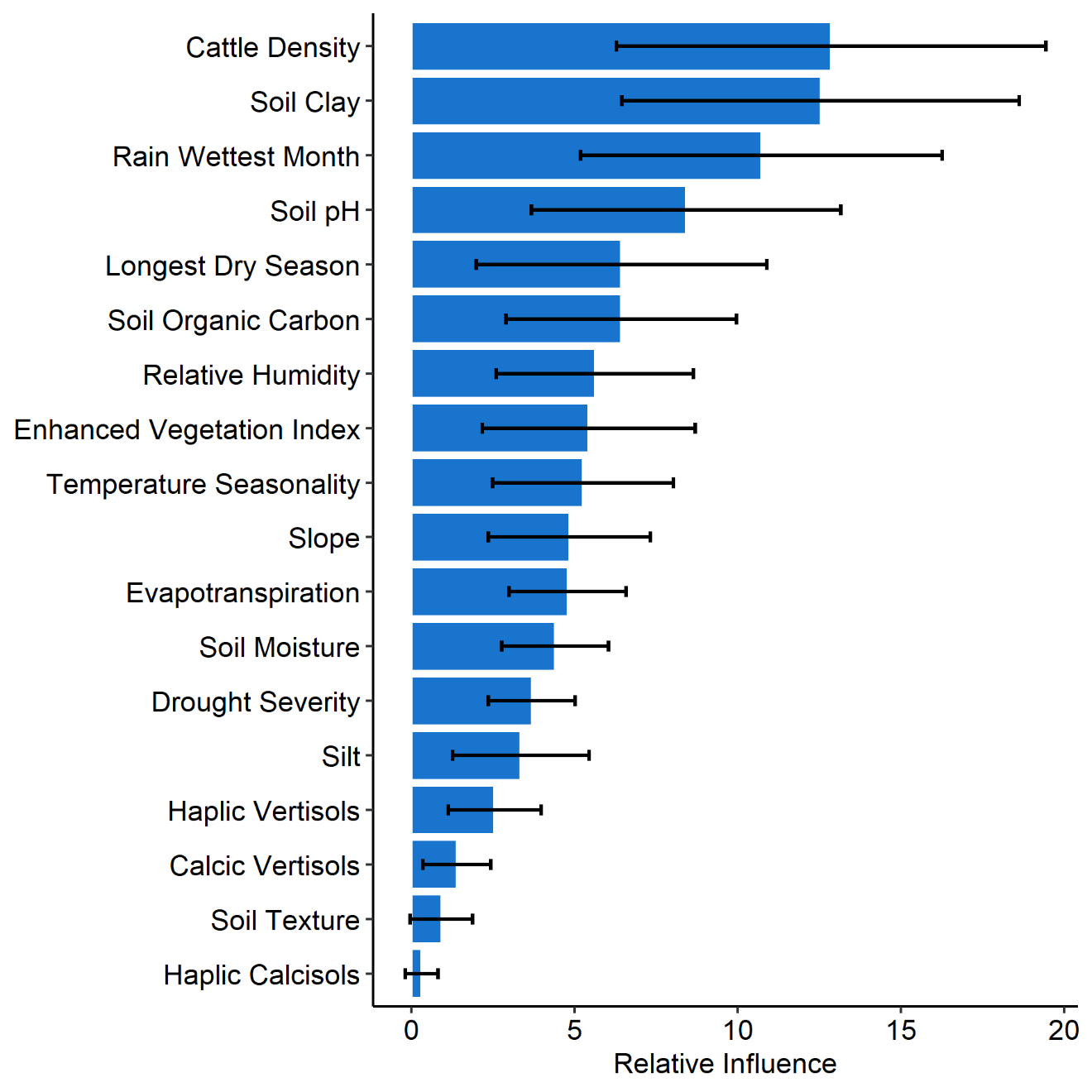


***Fig S3: Variable relative influence defining the ecological niche for anthrax outbreaks following the projection of the southern Kenya model into the whole of Kenya. Error bars represent variability across an ensemble of 100 BRT experiments.*** *This figure was generated using R software version 4.2.2. at http://cran.r-project.org.*
