# Supplementary material for: Anthrax hotspot mapping in Kenya support establishing a sustainable two-phase elimination program targeting less than 6% of the country landmass
Source: Sci Rep. 2022 Dec 15;12:21670. doi: 10.1038/s41598-022-24000-3 (PMC9755300; doi:10.1038/s41598-022-24000-3)
Supplement: Supplementary file 4 — Supplementary Figure S4. [file 41598_2022_24000_MOESM4_ESM.docx]

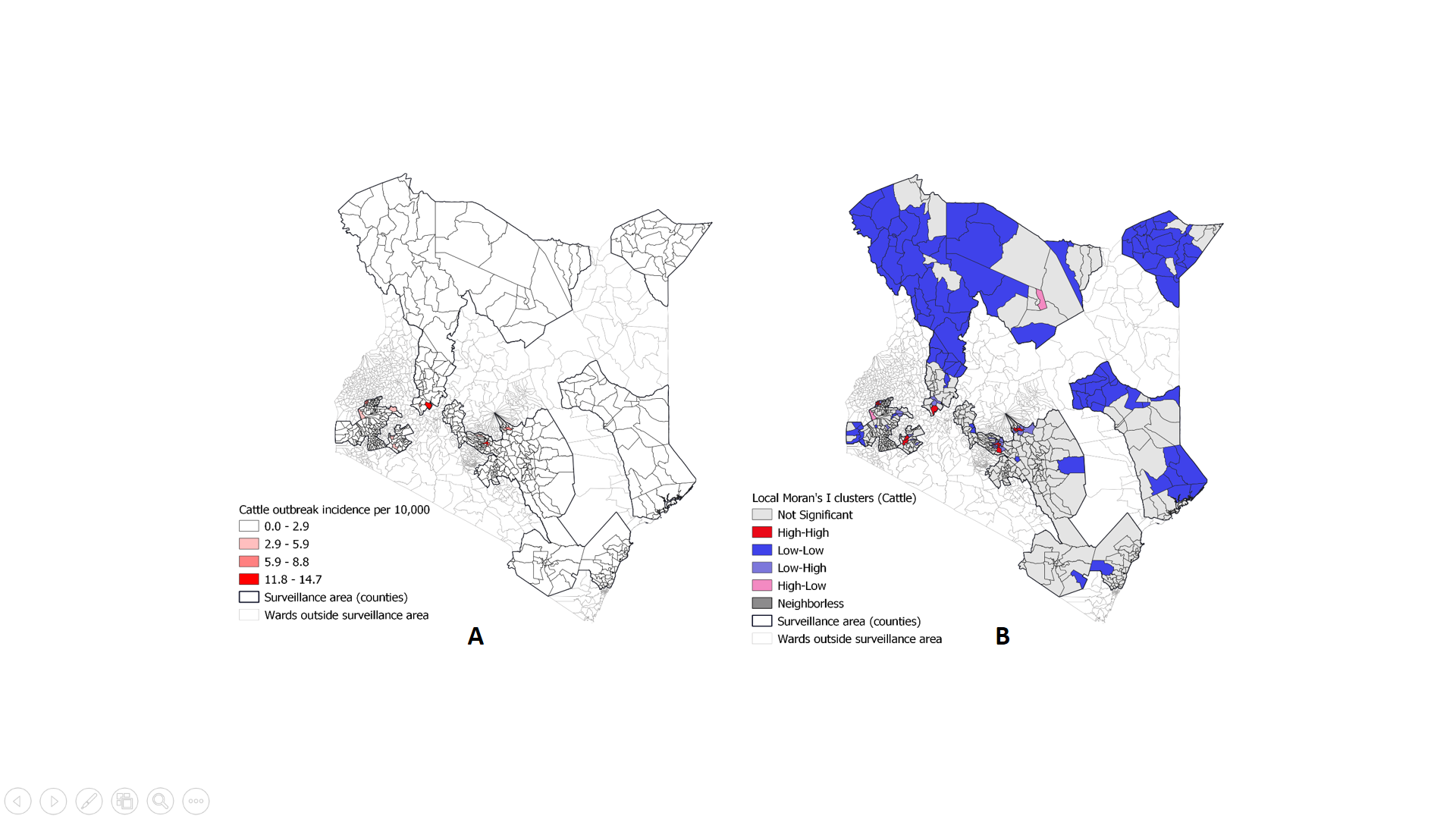


***Figure S4. A: Estimate of cattle outbreaks per 10,000 head of cattle using empirical Bayes smoothing. B: Local Moran’s I based spatial clusters and outliers of anthrax outbreak incidence per 10,000 head of cattle per ward using empirical Bayes smoothed estimates.*** *These figures were generated using R software version 4.2.2. at http://cran.r-project.org.*
