# Supplementary material for: Anthrax hotspot mapping in Kenya support establishing a sustainable two-phase elimination program targeting less than 6% of the country landmass
Source: Sci Rep. 2022 Dec 15;12:21670. doi: 10.1038/s41598-022-24000-3 (PMC9755300; doi:10.1038/s41598-022-24000-3)
Supplement: Supplementary file 5 — Supplementary Information 1. [file 41598_2022_24000_MOESM5_ESM.docx]

**Supplementary Text 1**

**Ecological niche model evaluation**

Since we first set aside 25% of the historical data to evaluate the projected Kenya model that used historical data to test model accuracy, the AUC was 0.77, similar to the original study. The Youden index cutoff for this study was a value of 0.52. In the second step, we examined the model accuracy of the same model using independent data composed of anthrax outbreaks data collected between 2017 and 2020. The percent of new anthrax outbreak locations predicted by dichotomized models shown in Fig 3 were as follows: For the southern Kenya model, a total of 88% of all new anthrax outbreaks fell in areas defined by the BRT models in our previous work. For the models predicting all of Kenya, 87% of all new anthrax outbreaks fell in areas defined by the BRT models, resulting in a validation AUC of 0.79.
